# Biological Collections: Chasing the Ideal

**Published:** 2016

**Authors:** P. A. Kamenski, A. E. Sazonov, A. A. Fedyanin, V. A. Sadovnichy

**Affiliations:** M.V. Lomonosov Moscow State University, Leninskie Gory 1, 119991, Moscow, Russia

## Abstract

This article is based on the results of an analysis of existing biological collections in Russia and abroad set up in the framework of the project “Scientific Basis of the National Biobank –Depository of Living Systems” by M.V. Lomonosov Moscow State University [1].

## DISCUSSION

**What is a “biological collection”?** Today, the terms “biological collection” and “biobank” are not interchangeable. A biological collection is typically defined as a systematized repository of a combination of any biological material type specimens. The term “biobank” is usually used only for collections of human biospecimens [2]. Hence, the term “biological collection” is broader than the term “biobank”: so, the former term will be used hereinafter in this study.

Based on the definition given above, several types of biological collections can be distinguished, depending on the types of biological material stored and the storage methods.

**Cryogenic collections **with material stored in frozen state are most often meant when talking about biological collections. These collections are created for long-term storage of biospecimens that are always supposed to retain their viability and normal functioning after being thawed. Cryogenic collections are typically used to store cells rather than entire organisms (of course, unicellular organisms are an exception). This storage method is applicable to cells of organisms of all life kingdoms on Earth. The cryostorage protocols are elaborated to the least extent for higher plant cells, which lose their viability relatively quickly during the freeze/thaw cycle because of their cytological features [3]. Nevertheless, these cells can also be successfully stored in cryogenic collections. Furthermore, nucleic acids extracted from living organisms can also be stored in frozen form; hence, the corresponding collections are also supposed to be classified as cryogenic collections.

**“Classical” collections **are another type of biological collections. First of all, they include collections of zoological museums and herbaria. Classical collections were the first collections of biological material in the world; some of them go back over two centuries. Recently, it has become clear that these collections of biological material, in addition to being used to study biodiversity, are a valuable source of DNA that can be extracted from the specimens stored and analyzed using the methods of molecular genetics [4]. This enables large-scale molecular phylogenetic studies for big samples amounts, which yield more statistically significant results.

When talking about biological collections, **collections of biological information **are often not included. This category is extremely important for the development of science and technology and mainly includes computer databases containing information about the primary, secondary, and tertiary structures of biological molecules. Such databases as GenBank (nucleotide sequences of the genomes of various organisms, http://www.ncbi.nlm. nih.gov/genbank/) and the Protein Data Bank (tertiary structures of proteins, http://www.rcsb.org/ pdb/home/home.do) can be mentioned as examples. The main distinctive feature of collections of biological information is their global character, which means that they can be freely accessed from any location via the Internet.

**Why do we need biological collections?**

In addition to the types of specimens stored, the purpose of biocollections can be viewed as a criterion for their classification.

Most collections that currently exist in the world are **research collections**. This term is used for sets of biological specimens that are stored in research laboratories and are needed in daily routines. Types of specimens in these collections may vary largely, from individual biological molecules to entire organisms. Research collections typically are small; however, they are today the main contributors to the development of fundamental life sciences and allow scientists to carry out comparative research. It should be mentioned that such collections can also be extensive. For example, collections of natural history museums and herbaria often comprise several million specimens and are actively used in research. The previously mentioned global collections of biological information are also used as research collections.

**Commercial collections **(i.e., collections of biological material that is intended for sale to external consumers) have recently acquired wide usage. In fact, these collections began emerging several decades ago, but the specimens stored were intended exclusively for research. Several collections of baker’s yeast strains can be given as an example [5]. The later created commercial collections most typically store donated human biological material (sex cells, blood cells, etc.) and belong to private institutions. Consumers of specimens from commercial collections may be both individuals (e.g., those using *in vitro* fertilization) and pharmaceutical companies that use human cell material for preliminary clinical trials of drugs.

Finally, **“state” collections **(i.e., the ones that are created and maintained for the sake of the state) need to be mentioned. First of all, this category includes collections intended for the preservation of biodiversity. Zoological and botanical gardens (although they are used both for research and common cultural purposes), as well as nature reserves and wildlife refuges, are examples of such collections. Collections for the preservation of biodiversity are not necessarily state-sponsored; they can be created by the initiative of an academic community, such as the Frozen Ark International Consortium that has been organized on the basis of several dozen research laboratories storing frozen cell material collected from rare and endangered species [6]. Along with preservation of biodiversity, state collections can be created to optimize the efficient use of biological resources. For example, the Natural Product Repository at the National Institute of Health (USA) [7] has over 100,000 extracts from various animals and plants that are used to search for novel biologically active agents. The All-Russian Collection of Industrial Microorganisms is an example of this type of collections in Russia; it is the only organization thus far that has been given the status of a national bioresource center by the state [8]. This collection includes microorganisms that are critically important for biotechnology. We would also like to mention such All-Russian collections as the All-Russian Collection of Microorganisms and the Russian Cell Culture Collection.

One should remember that preservation of biodiversity is also related to national security. First and foremost, such collections are meant for the preservation of producer cells for industrial and medical biotechnology, as well as bio-objects for cattle breeding and crop production. Under crisis conditions, Russian industry and agriculture will be thrown back decades if there are no Russian collection repositories.

**Russian and foreign biocollections.** *Table* summarizes the data on the largest Russian collections of biological materials.

**Table:** The largest Russian biological collections (in descending order of the amount of repository items) according to the official websites of the corresponding organizations

Collection	Organization	Type of specimens stored	Amount of specimens stored	Purposes of specimens stored	Pipelines
Collection of Zoological Institute	Zoological Institute, RAS	Animal biomaterial (non-living)	60,000,000	Research, museum affairs	https://www.zin.ru/Collections/
Zoological Museum, MSU	Moscow State University	Animal biomaterial (non-living)	10,000,000	Research, museum affairs	http://zmmu.msu.ru/
Collection of Botanical Institute	Botanical Institute, RAS	Dried plant and small amounts of plant cell cultures	7,000,000	Research	http://www.binran.ru/collections/
Herbarium, MSU	Moscow State University	Dried plants	1,000,000	Research	http://herba.msu.ru/russian/departments/herbarium/General_Information/Collection/
N.I. Vavilov collection of cultivated plant seeds	N.I. Vavilov Institute of Plant Genetic Resources, RAS	Cultivated plant seeds	300,000	Preservation of the beneficial biodiversity, agriculture	http://vir.nw.ru/otd_r.htm#dept
All-Russian collection of microorganisms	Institute of Biochemistry and Physiology of Plants and Microorganisms, RAS	Frozen microorganisms	20,000	Delivery ofspecimens to the external consumers, research	http://ibpm.ru/index.php?option=com_content&view=article&id=249:vkm&-catid=4&Itemid=15
Biological Resource Center “Russian National Collection of Industrial Microorganisms”	All-Russian Institute of Genetics and Selection of Industrial Microorganisms	Frozen microorganisms	20,000	Biotechnology, research	http://vkpm.org/o-bioresursnom-tsentre/
Russian cell culture collection	The collection is stored in 9 different organizations. Principal organization: Institute of Cytology, RAS	Frozen human, animal and plant cells	3,000	Delivery of specimens to the external consumers, research	http://www.cytspb.rssi.ru/rkkk/rkkk_ru.htm

As it has been mentioned previously, the main objectives of creating and maintaining biocollections include preservation, research, and the beneficial use of biodiversity. Speaking about Russian biological collections, the research function is the best-developed. The *Table* demonstrates that the vast majority of large Russian collections perform research activities and annually publish hundreds of studies focused on a thorough investigation of the specimens stored.

The situation with biodiversity preservation is more complex. Under the current state of technology development, the problem of biodiversity preservation needs to be solved at two levels; namely, the organism and cell levels. At the former level, work is being carried out in zoological and botanical gardens and nature reserves; Russia is in a rather strong position here. As of 2014, there were 104 nature reserves in Russia [9]; this number is significantly larger than that in any other country. On the other hand, it cannot be denied that nowadays the traditional measures of preservation of rare and endangered living species need to be supplemented with high-tech measures; that is, the storage of cell material harvested from these organisms in cryogenic collections. This aspect contributes most significantly to Russia’s lag behind other industrialized countries. Whereas the international consortia preserving cell material harvested from rare species have been operating abroad for an appreciably long time (e.g., the aforementioned Frozen Ark), such work in Russia has just started.

The beneficial use of Russian biological resources is also far from perfect. Living systems are practically used mainly in biotechnology and medicine. When it comes to biotechnology, it is noteworthy that the performance of Russian biocollections is rather active. This mostly relates to the collections of microorganisms (Russian National Collection of Industrial Microorganisms, All-Russian Collection of Microorganisms, several small collections of microalgae) that have been successfully implementing the results of their activity for a long time by creating and optimizing strains producing various compounds. On the other hand, the use of biocollections in medicine in Russia is currently at its lowest level. In Europe and the U.S., 5 years ago there were already several dozen both global and specialized large collections of human cell material [10] and their number has increased since then. The specimens in these collections are actively used in biomedical research and pilot projects. Today, when medical cellular technologies develop in explosive fashion, these collections become particularly important. Finally, it cannot go unmentioned that there are no large collections related to cattle breeding in Russia. Undoubtedly, this fact significantly slows the development of agriculture as a key sector of the Russian economy.

Having analyzed the current state of biological collections, we are certain that these collections represent a tremendous research and technological capability which is currently not being exploited for several reasons. So, **what are the challenges that Russian specialists who organize and maintain biological collections face today**? First of all, it is worth mentioning once again that the cryogenic collections of state status aimed at the preservation and beneficial use of the biodiversity of Russia are poorly developed (see text above).

A serious problem affecting modern biocollections (not only in Russia but worldwide) is that they are disaggregated. This is especially true for the small research collections that most research laboratories have, as was mentioned previously. It is quite typical that scientists maintaining and working with their local collections do not notify the research community at large of the specimens stored in their collections. This happens either because the scientists lack the resources to do so, or sometimes because they are oblivious to the fact that their material can be extremely useful to their colleagues from other organizations. Meanwhile, virtual integration of research (and not only research) collections into a consolidated information system would be extremely efficient today, as large-scale studies using big amounts of samples of different biological objects become increasingly important. Understanding of this problem also exists at the state level. In 2014, a working group was established by the Ministry of Education and Science of the Russian Federation to elaborate approaches to creating national biological resource centers on the basis of the existing biological collections. One of the main tasks of the working group was to perform a global auditing of existing biological collections in Russia, creating an integrated database that would include these collections, and elaborating a mechanism for reorganizing them into national bioresource centers. In 2015, a similar initiative was proposed by the Russian Academy of Sciences and the Federal Agency for Scientific Organizations by establishing a working group to maintain and develop bioresource collections. Its objectives include auditing research collections and elaborating recommendations for their centralization, standardization, and accessibility. Beyond any reasonable doubt, the activity of these working groups will increase the output of Russian scientists.

It should be mentioned that it is almost impossible to obtain funding for work related to direct maintenance of biocollections. Scientific foundations and the research programs of the ministries do not classify this field as research and usually do not consider the applications submitted. Neither does this activity fall within state assignments for higher educational institutions and research institutes. As a result, biocollections either get whatever funds remain or are maintained out of sheer enthusiasm (those are the two most common terms to describe the situation around the maintenance of biocollections).

Finally, it should not go unmentioned that there is virtually no legal framework for Russian biological collections. Meanwhile, establishment of a legal framework for working with biocollections is absolutely critical, mainly due to the close connection between biological collections and the concept of “national biological resources.” This concept is similar to the term “biological diversity” but is broader, since it includes genetic resources of the country’s population, biotechnological resources, and natural resources. Bioresources fall under strict legal regulations all over the world; however, regulation in Russia is weaker than in other industrialized countries. Moreover, the import and export of biological material for scientific purposes is unregulated. Because of the lack of accurate procedures, exchange of biomaterial –one of the key aspects of international scientific collaboration –either is impossible (which closes the door on Russian researchers’ participation in many important and prestigious scientific consortia) or forces scientists to act as smugglers, which is unacceptable.

Furthermore, no laws that regulate work with human biomaterial exist yet (in 2015, the Law On Biomedical Cell Technologies was adopted only in the first reading in the Russian State Duma); the situation around the regulation of genetic modification of living organisms is not fully clear (the corresponding draft bill is been consideration by the Russian State Duma since 2015). On the other hand, Russian government agencies now understand the importance of elaborating such documentation; work towards it is under way, and members of the scientific community are involved. However, the entire state apparatus, including its executive and legislative branches, needs to consolidate in order to overcome the existing administrative barriers.

Based on the aforementioned, we would like to suggest measures to be taken in order to use biological collections in the Russian Federation with maximum efficiency:

1. Auditing the existing collections;

2. Sharing best practices in the field of biocollections; elaborating uniform protocols to work with biospecimens of the same type;

3. Creating a single database that would contain information about the maximum possible number of collections; in the long run, establishing a national information and analysis system;

4. Establishing a research center based on large collections under state assignment; establishing a national network of biocollections;

5. Collaboration between members of the scientific community involved in dealing with biocollections and state agencies to develop adequate measures for regulating the activities of biocollections and the related fields of science and technology; and

6. Creating a global “biocollection information space.”
